# Imidazole as a parent π-conjugated backbone in charge-transfer chromophores

**DOI:** 10.3762/bjoc.8.4

**Published:** 2012-01-05

**Authors:** Jiří Kulhánek, Filip Bureš

**Affiliations:** 1 Institute of Organic Chemistry and Technology, Faculty of Chemical Technology, University of Pardubice, Studentská 573, Pardubice, CZ-53210, Czech Republic

**Keywords:** charge transfer, chromophore, conjugation, donor–acceptor system, imidazole

## Abstract

Research activities in the field of imidazole-derived push–pull systems featuring intramolecular charge transfer (ICT) are reviewed. Design, synthetic pathways, linear and nonlinear optical properties, electrochemistry, structure–property relationships, and the prospective application of such D-π-A organic materials are described. This review focuses on Y-shaped imidazoles, bi- and diimidazoles, benzimidazoles, bis(benzimidazoles), imidazole-4,5-dicarbonitriles, and imidazole-derived chromophores chemically bound to a polymer chain.

## Introduction

**Figure 1:**
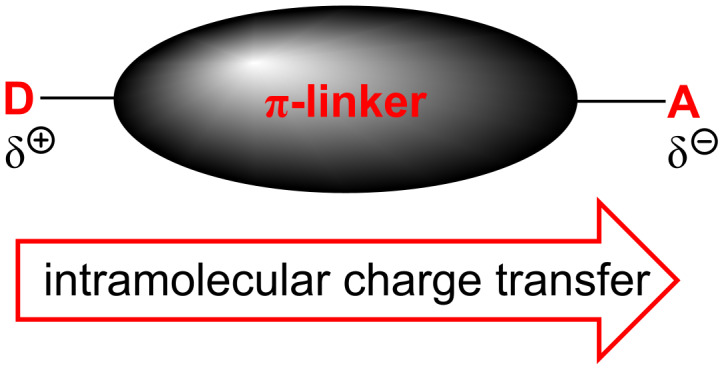
Schematic representation of organic D-π-A system featuring ICT.

Over the past three decades, great progress has been made in the development and the investigation of new organic push–pull systems. In contrast to inorganic materials, the advent of dipolar (hetero)organic materials with readily polarizable structure was stimulated by their relative ease of synthesis, well-defined structure, chemical and thermal robustness, possibility for further modification, and facile property tuning. Hence, heteroaromatic push–pull chromophores have been targeted and investigated as active components of optoelectronic devices, organic light-emitting diodes (OLED), photovoltaic cells, semiconductors, switches, data-storage devices, etc [1–3]. A typical one-component organic D-π-A chromophore consists of a π-conjugated system end-capped with strong electron donors D (e.g. NR_2_ or OR groups) and strong electron acceptors A (e.g. NO_2_ or CN groups). This D-π-A arrangement assures efficient intramolecular charge transfer (ICT) between the donor and acceptor moieties and generates a dipolar push–pull system featuring low-energy and intense CT absorption (Figure 1). The polarizability and the respective optical linear and nonlinear (NLO) properties of these systems depend primarily on their chemical structure, in particular, the electronic behavior of the appended donors and acceptors and the character and length of the π-conjugated linker [4–7].

**Figure 2:**
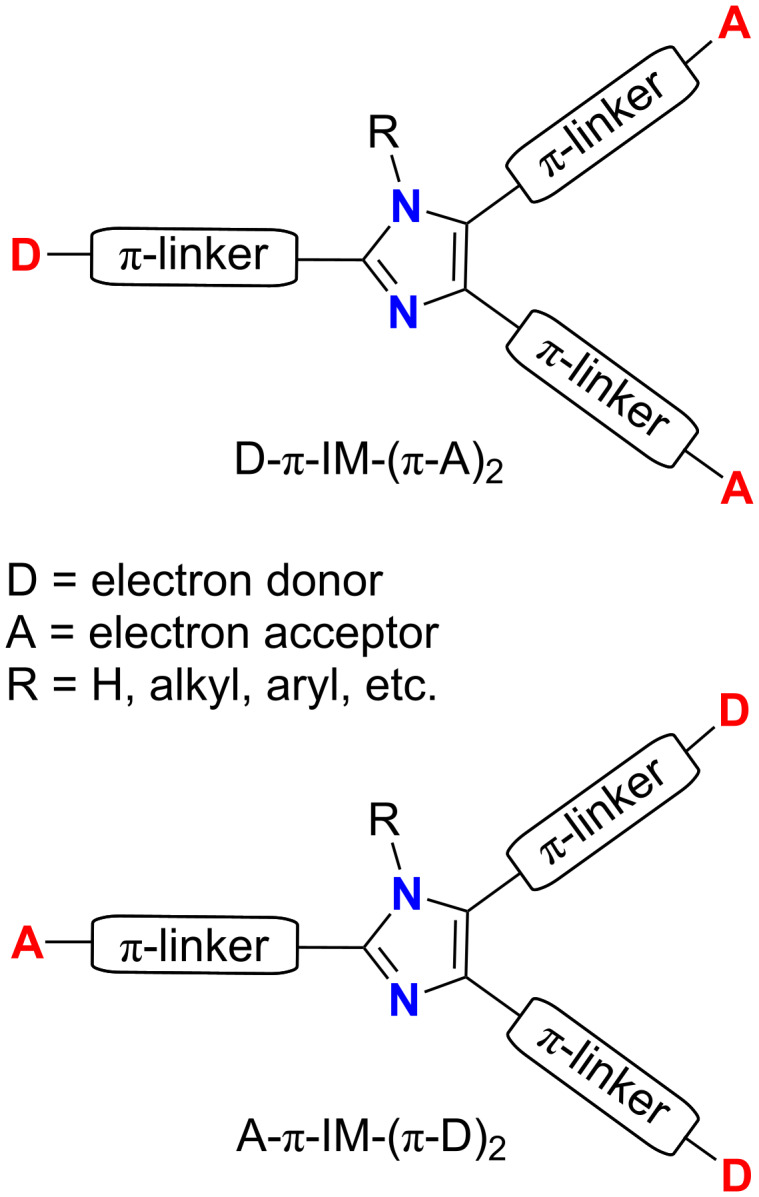
Two principal orientations of the imidazole-derived charge-transfer chromophores.

Recently, it was also recognized that push–pull systems applicable as organic materials should possess high chemical and thermal robustness, good solubility in common organic solvents, and should be available in reasonable quantities. Hence, various five- and six-membered heterocycles were utilized as suitable π-conjugated chromophore backbones. Moreover, heteroatoms may act as auxiliary donors or acceptors and improve the overall polarizability of the chromophore. In this respect, five-membered diazoles, in particular imidazole, seem to be suitable parent π-conjugated backbones. Imidazole possesses two nitrogen atoms of different electronic nature, represents a robust and stable heterocycle, and can easily be synthesized and further functionalized at positions C2, C4, and C5 in addition to N1. On the imidazole backbone, two principal orientations of the substituents are possible, and these are most frequently used to generate Y-shaped chromophores as shown in Figure 2. The donor appended through an additional π-linker to the imidazole C2, completed with two peripheral acceptors linked at the imidazole C4/C5 positions, generates the first class of chromophores (D-π-IM-(π-A)_2_ systems). The second class (A-π-IM-(π-D)_2_ systems) possesses one acceptor and two donors in the reversed orientation. A nonsymmetrical orientation of the donors and acceptors is scarce, most likely due to a more difficult synthesis.

The purpose of this article is to review the recent progress in the design, development, and investigation of imidazole-derived charge-transfer chromophores. Synthetic pathways, linear and nonlinear optical properties, electrochemistry, and the prospective application of such organic materials are described. Metal complexes and metal sensitizers are not covered in this review.

## Review

### Synthesis of imidazole-derived chromophores

**Scheme 1:**
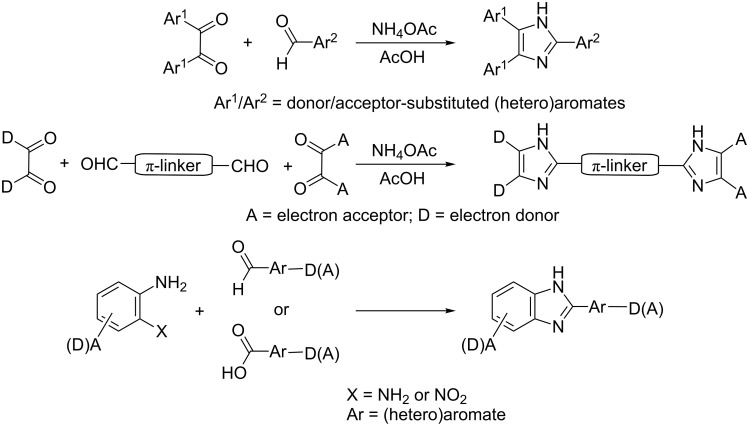
Common synthetic approach to triarylimidazole-, diimidazole-, and benzimidazole-derived CT chromophores [11–26].

A condensation of α-diketones and aldehydes in the presence of ammonia or ammonium salts (Debus–Radziszewski synthesis) is one of the oldest, most versatile, and most frequently employed methods used for the construction of imidazole (glyoxaline) derivatives [8–10]. This simple synthetic pathway is also widely employed for the construction of variously substituted 2,4,5-triarylimidazole-derived chromophores (lophines), as shown in Scheme 1 [11–15].

A similar synthetic strategy is used for the construction of diimidazole-type push–pull systems bearing two imidazole rings, which serve as donor and acceptor moieties [16–19]. A sequential construction of the chromophore backbone by modern cross-coupling reactions represents another synthetic approach used for the synthesis of superior diimidazole chromophores [20]. Benzimidazole D-π-A derivatives are a well-investigated class of charge-transfer chromophores. Although many synthetic approaches are known to date [10,21–22], the most popular ones involve the condensation of appropriately substituted arylenediamines or *o*-nitroanilines with an aldehyde or carboxylic acid, as well as Debus–Radziszewski synthesis as shown in Scheme 1 [23–26].

**Scheme 2:**
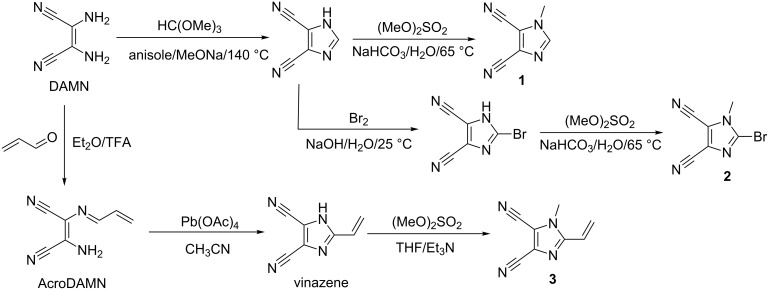
Syntheses of important 4,5-dicyanoimidazole derivatives **1**–**3** [27–30].

Since the discovery and the first synthesis of 4,5-dicyanoimidazole was reported by Woodward [27], this imidazole derivative became a popular moiety with moderate acceptor power. Starting from diaminomaleonitrile (DAMN), the simple synthesis of 1-methylimidazole-4,5-dicarbonitrile (**1**) is outlined in Scheme 2 along with the preparation of 2-bromo-1-methylimidazole-4,5-dicarbonitrile (**2**) and 1-methyl-2-vinylimidazole-4,5-dicarbonitrile (methylvinazene, **3**) [28–30]. These derivatives were recently utilized as suitable precursors for the construction of various CT chromophores (see below).

### Y-shaped imidazole-derived chromophores

**Figure 3:**
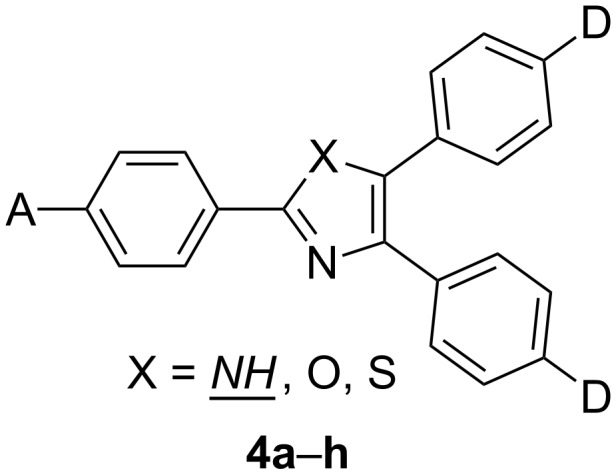
Donor–acceptor triaryl push–pull azoles **4a**–**h** [31–32].

Triarylimidazoles (lophines) and derivatives with larger π-linkers represent the simplest D-π-IM-(π-A)_2_ and A-π-IM-(π-D)_2_ push–pull systems. An initial effort to synthesize and apply azole derivatives as CT chromophores and to study their optical (non)linearities can be ascribed to Moylan, Miller, and co-workers as early as 1993 [31–32]. Donor–acceptor-substituted imidazoles, oxazoles, and thiazoles were synthesized, and their properties were compared within the individual series of substituents as well as across the three heterocyclic rings (Figure 3, Table 1). These A-π-IM-(π-D)_2_ systems possess exceptional thermal stabilities, respectable dipole moments, and significant nonlinearities. It was found that the chromophore nonlinearity depends primarily on the type of substituents A/D and secondarily on the nature of the conjugating heterocyclic ring (Table 1).

**Table 1:** Linear (λ_max_) and nonlinear (β) optical properties of triarylimidazoles **4a**–**h** (X = NH) [31].

Comp. X = NH	A	D	λ_max_^a^ [nm]	μ [D]	β^b^ [10^−30^ esu]

**4a**^c^	NO_2_	OMe	412	6.4	19.9
**4b**^d^	C≡CPhNO_2_	OMe	400	8.1	69.1
**4c**^d^	SO_2_Ph	OMe	362	8.0	10.1
**4d**^c^	NO_2_	N[*c*-(CH_2_)_5_]	438	7.2	45.5
**4e**^c^	NO_2_	OCH_2_(C_2_H_5_)CHC_4_H_9_	416	6.3	24.5
**4f**^c^	SO_2_C_4_F_9_	OCH_2_(C_2_H_5_)CH_2_CH_4_H_9_	384	6.5	13.9
**4g**^d^	NO_2_	C≡CPhOMe	344	8.0	53.2
**4h**^c^	NO_2_	N[*c*-(CH_2_)_6_]	476	8.3	78.7

^a^Position of the longest-wavelength absorption maxima; ^b^molecular first-order hyperpolarizability measured by EFISH experiments at 1064 or 1907 nm; ^c^measured in CHCl_3_; ^d^measured in 1,4-dioxane.

**Figure 4:**
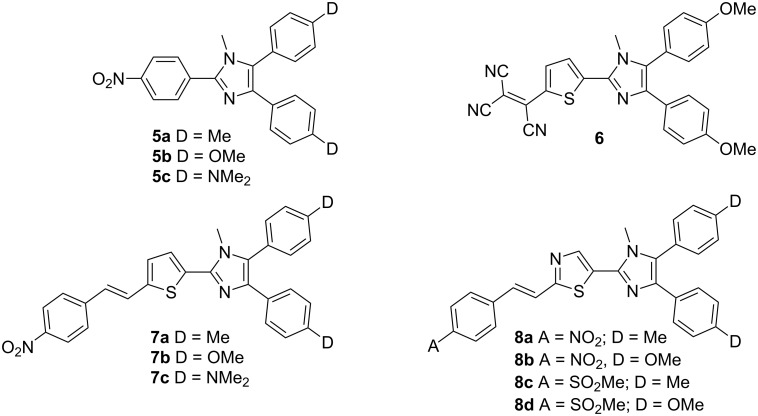
Y-shaped CT chromophores with an extended π-conjugated pathway and various donor and acceptor substitution patterns [16,33–35].

More recently, Bu and co-workers also contributed significantly to the field of imidazole-derived CT chromophores for NLO. The first class of studied compounds **5a**–**c** resembles those chromophores reported by Moylan et al.: The parent π-conjugated backbone of *N*-methyllophine end-capped with two donors and one acceptor [33]. Bu’s further efforts were focused on (i) the incorporation of an additional, readily polarizable heterocycle, such as thiophene or thiazole; (ii) the improvement of the electron-withdrawing ability of the used acceptor; and (iii) the elongation of the π-conjugated pathway. Thus, the first series of chromophores (**5a**–**c**) was completed with the thiophene-derived system **6** [14] with a tricyanovinyl acceptor moiety and chromophores **7a**–**c** [33] featuring a thiophene π-linker and a nitrostyryl acceptor. The molecular structures of compounds **6** and **5c** were also confirmed by X-ray analysis [34]. The last series of investigated compounds involved donor 4,5-disubstituted imidazoles **8a**–**d** [35] with acceptors at C2 linked through a thiazole-styryl π-linker (Figure 4). Whereas the series **5a**–**c** and **7a**–**c** showed promising optical nonlinearities, high thermal stability, excellent solubility, and good transparency, molecules **8a**–**d** were investigated as two-photon absorbing chromophores (Table 2). Bu and co-workers also investigated the fluorescence properties of this family of imidazoles [36].

**Table 2:** Selected properties of chromophores **5**–**8** [33–35].

Comp.	A	D	λ_max_^a^ [nm]	μβ^b^ [10^−48^ esu]	δ^c^ [10^−50^ GM]

**5a**	–	Me	–	145	–
**5b**	–	OMe	–	130	–
**5c**	–	NMe_2_	–	360	–
**6**	–	–	–	-	–
**7a**	–	Me	432	945	–
**7b**	–	OMe	436	475	–
**7c**	–	NMe_2_	463	590	–
**8a**	NO_2_	Me	422	–	650 (720 nm)
**8b**	NO_2_	OMe	433	–	1050 (720 nm)
**8c**	SO_2_Me	Me	398	–	1400 (740 nm)
**8d**	SO_2_Me	OMe	410	–	1700 (760 nm)

^a^Measured in CHCl_3_; ^b^scalar product of the dipole moment and the molecular first-order hyperpolarizability measured in CHCl_3_ by EFISH experiments at 1907 nm; ^c^the 2PA cross section measured in CHCl_3_ by Z-scan technique (1 GM = 1 × 10^−50^ cm^4^·s·photon^−1^) at the given 2PA wavelengths.

**Figure 5:**
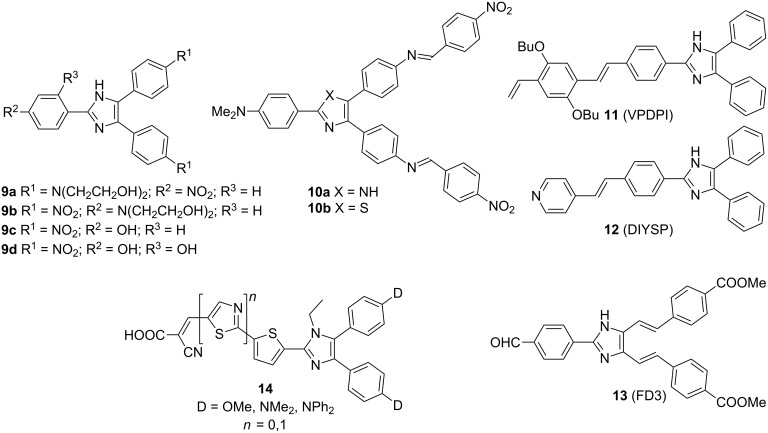
Molecular structures of chromophores **9**–**14** [13,15,37–41].

In addition to the work of Moylan and Bu, several other groups, mainly from Asia, reported the synthesis and application of Y-shaped imidazole-derived CT chromophores. Wang and co-workers investigated simple tripodal chromophores **9a**–**d** with nitro, dialkylamino, and hydroxy groups as acceptor and donors [13]. Whereas the imidazole- and thiazole-based chromophores **10a**,**b** possess two extended π-linkers with the imino spacers at the imidazole C4/C5 and nitro and dimethylamino groups as acceptor and donor [15], chromophore **11** (VPDPI) represents a polarizable blue-light-emitting material [37]. The newly synthesized chromophores were investigated in terms of their absorption and emission properties, molecular first-order hyperpolarizability β, measured by solvatochromic method at 1907 nm, and thermal stability determined by TGA or DTA (Figure 5, Table 3). Imidazoles **12** (DIYSP, δ = 41 GM, [38–39]) and **13** (FD3, δ = 1556 GM, [40]) were developed as two-photon absorbing and fluorescent A-π-A’ chromophores, which undergo photopolymerization or can be applied as fluorescent sensors for (homo)cysteine. Donor 4,5-disubstituted imidazole derivatives **14** bearing a cyanoacrylic moiety connected to imidazole C2 by thiophene or thiazole π-linkers were recently utilized as dye-sensitized solar cells with an efficiency up to 6.3% [41].

**Table 3:** Linear and nonlinear optical properties and thermal stabilities of chromophores **9**–**11** [13,15,37].

Comp.	λ_max,abs_^a^ [nm]	λ_max,em_^b^ [nm]	β^c^ [10^−30^ esu]	*T*_D_^d^ [°C]

**9a**	403	–	68.42	300
**9b**	388	–	36.92	282
**9c**	345	–	89.01	299
**9d**	380	–	54.65	265
**10a**	358	–	40.66	227
**10b**	417	–	17.31	289
**11**	380	465 (Φ = 0.61)	–	367

^a^The position of the longest-wavelength absorption maxima measured in 1,4-dioxane (**9**), MeOH (**10**), and EtOH (**11**); ^b^the position of the longest-wavelength emission maxima measured in EtOH; ^c^molecular first-order hyperpolarizability measured by solvatochromic method at 1907 nm; ^d^determined by TGA or DTA.

Several similar classes of imidazole-derived push–pull compounds can be found in the literature. They were mainly investigated in terms of their synthesis and basic (non)linear optical properties [42–46].

**Figure 6:**
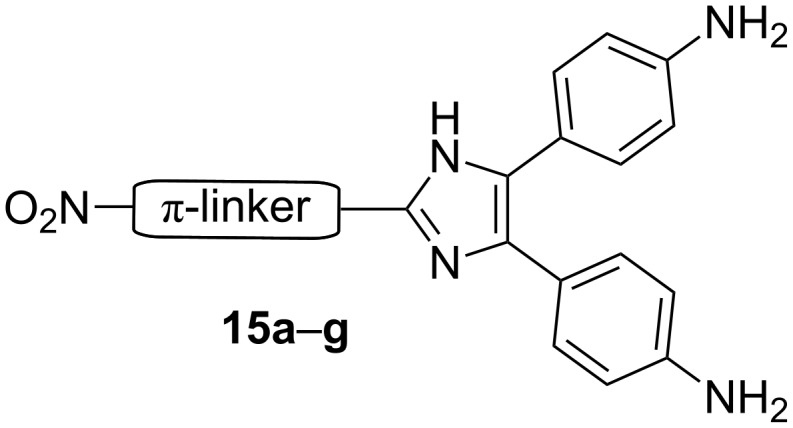
General structure of 4,5-bis(4-aminophenyl)imidazole-derived chromophores **15a**–**g** with various π-linkers [16–17,47].

Wu et al. utilized 4,5-bis(4-aminophenyl)imidazole as a suitable donor moiety for the construction of the nitro C2-substituted imidazole push–pull systems with extended and varied π-conjugated pathway **15a**–**g** [16–17,47]. The π-linker comprises 1,4-phenylene (C_6_H_4_), thiophen-2,5-diyl (C_4_H_2_S), ethenylene, and azo subunits (Figure 6, Table 4). An evaluation of the NLO data in Table 4 clearly shows that an elongation of the π-linker by polarizable subunits, such as a double bond or a thiophene, increases the measured second-order hyperpolarizability β significantly and also shifts the CT band bathochromically.

**Table 4:** Properties of 4,5-bis(4-aminophenyl)imidazole-derived chromophores **15a**–**g** [16–17,47].

Comp.	π-linker	λ_max_^a^ [nm]	μ [D]	β^b^ [10^−30^ esu]	*T*_D_ [°C]

**15a**	–(C_6_H_4_)–	405	7.7	17.19	300
**15b**	–(C_6_H_4_)–CH=CH–(C_6_H_4_)–	401	8.2	34.78	335
**15c**	–(C_6_H_4_)–N=N–(C_6_H_4_)–	461	8.2	41.82	344
**15d**	–(C_6_H_4_)–IM–[4,5-di–(C_6_H_4_)]–	384	10.9	50.91	377
**15e**	–(C_4_H_2_S)–	419	8.6	22.52	286
**15f**	–(C_4_H_2_S)–CH=CH–(C_6_H_4_)–	435	9.1	44.56	279
**15g**	–(C_4_H_2_S)–CH=CH–(C_4_H_2_S)–CH=CH–(C_6_H_4_)–	463	9.1	101.9	268

^a^Measured in THF; ^b^calculated by AM1/FF method (MOPAC).

**Figure 7:**
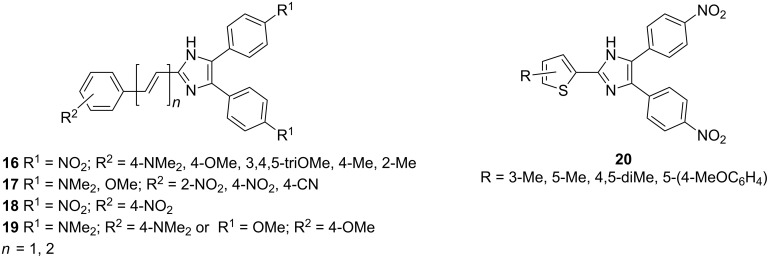
Various orientations of the substituents on the parent lophine π-conjugated backbone (**16**–**19**) and thiophene-substituted imidazoles **20** [11].

In 2009, our group also contributed to Y-shaped imidazole-derived chromophores [11]. We synthesized a library of substituted lophines **16**–**19** with four types of donor–acceptor orientations (Figure 7): D-π-IM-(π-A)_2_ (**16**), A-π-IM-(π-D)_2_ (**17**), A-π-IM-(π-A)_2_ (**18**), and D-π-IM-(π-D)_2_ (**19**). 4,5-Bis(4-nitrophenyl)imidazole was utilized as a suitable acceptor moiety and was further modified with a thiophene π-linker as an auxiliary electron donor (**20**). These basic push–pull imidazoles were mainly investigated in terms of their facile synthesis, spectral properties, and thermal stability.

**Figure 8:**
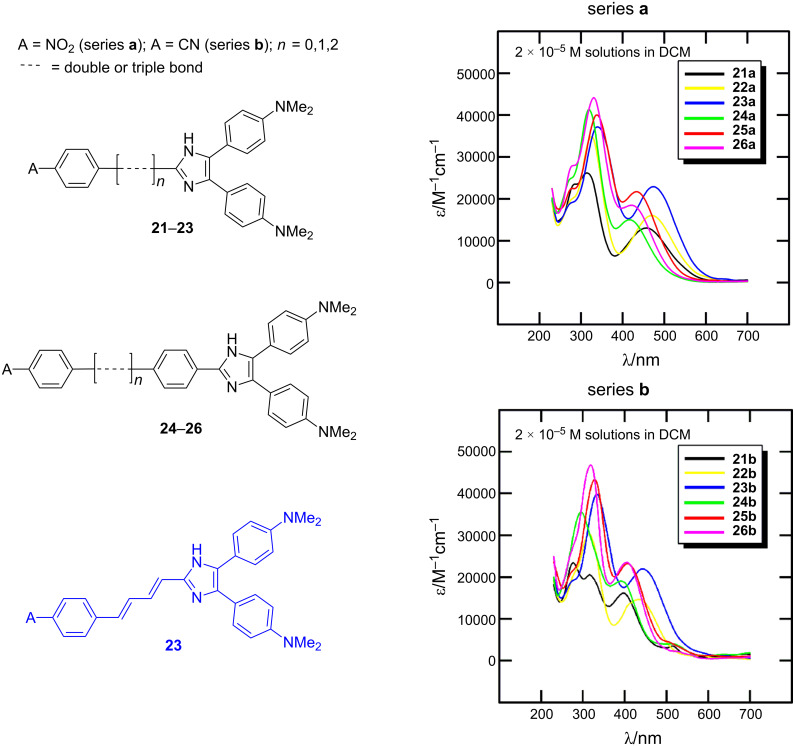
Structure and electronic absorption spectra of chromophores **21**–**26** [12].

The 4,5-bis[4-(*N*,*N*-dimethylamino)phenyl]imidazole unit, as in chromophores **17**, was further used for the construction of A-π-IM-(π-D)_2_ systems **21**–**26** with a systematically extended π-conjugated pathway (Figure 8; [12]). These chromophores were synthesized by Debus–Radziszewski synthesis (Scheme 1), as two series of compounds with different acceptors A (NO_2_ or CN groups), and were investigated by electrochemistry, UV–vis and IR spectroscopy (CN), and quantum-chemical calculations (Table 5). Considering all the above measured and calculated properties, we can deduce that the following structure–property relationships determine the extent of ICT: (i) The presence of a strongly conjugating acceptor (NO_2_/CN); (ii) the π-system length and structure; and (iii) the overall chromophore planarity. Hence, chromophores **23a**,**b** with fully planar, central 4-phenylbuta-1,3-dienyl π-linker, end-capped with 4,5-bis[4-(*N*,*N*-dimethylamino)phenyl]imidazole donor and nitro and cyano acceptors, feature good solubility in common organic solvents as well as the lowest measured electrochemical gaps *E*_p,a_−*E*_p,c_, the most bathochromically shifted CT bands (λ_max_), the lowest frequency of CN stretch (**23b**), and the highest calculated average second-order polarizabilities β within the studied series of compounds **21**–**26**.

**Table 5:** Properties of chromophores **21**–**26** [12].

Comp.	A	*n*	Bond^a^ [.....]	*E*_p,a_−*E*_p,c_^b^ [V]	λ_max_^c^ [nm (eV)]	ν (CN)^d^ [cm^−1^]	β^e^ [10^−30^ esu]	mp [°C]

**21a**	NO_2_	0	–	1.62	457 (2.71)	–	57.6	105–106
**21b**	CN	0	–	2.53	397 (3.12)	2221	36.8	123–125
**22a**	NO_2_	1	d	1.53	470 (2.64)	–	59.6	163–167
**22b**	CN	1	d	2.23	434 (2.86)	2220	46.2	142–144
**23a**	NO_2_	2	d	1.48	474 (2.62)	–	89.8	157–160
**23b**	CN	2	d	2.06	442 (2.81)	2218	66.9	161–163
**24a**	NO_2_	0	–	1.57	417 (2.97)	–	48.1	159–162
**24b**	CN	0	–	2.42	391 (3.17)	2223	39.9	170–173
**25a**	NO_2_	1	d	1.53	434 (2.86)	–	78.0	165–166
**25b**	CN	1	d	2.23	407 (3.05)	2219	41.7	165–168
**26a**	NO_2_	1	t	1.46	420 (2.95	–	84.6	262–264
**26b**	CN	1	t	2.26	405 (3.06)	2220	63.1	162–165

^a^d/t = double/triple bond; ^b^*E*_p,a_ and *E*_p,c_ are anodic and cathodic peak potentials measured by CV (potentials given vs. SCE); ^c^measured in CH_2_Cl_2_; ^d^frequency of the C≡N stretch (series **b**); ^e^calculated average second polarizability by AM1/FF (MOPAC).

The nonlinear optical properties of donor- and acceptor-substituted five-membered heterocycles, such as imidazole, oxazole, and thiazole, were also investigated by DFT calculations [48–49]. These theoretical results confirmed, in general, the experimental data and trends discussed above.

### Diimidazole-derived chromophores

**Figure 9:**
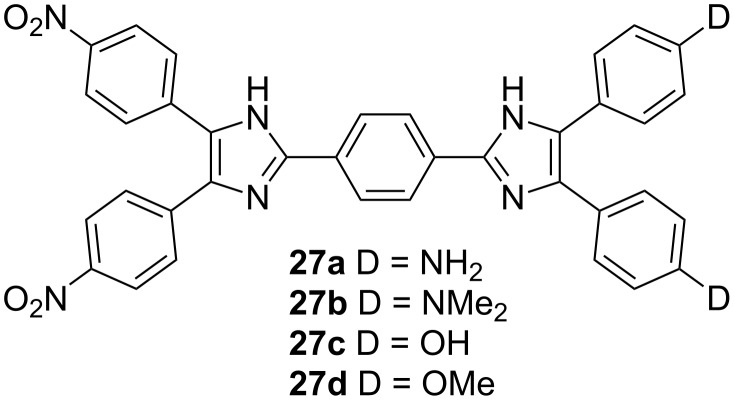
Typical D-π-A diimidazole CT chromophore [16–18,50–53].

The aforementioned charge-transfer chromophores **4**–**26** consist of a 1,2,4,5-tetrasubstituted imidazole ring, which may act as either a donor or acceptor moiety depending on the orientation of substituents. A π-conjugated backbone end-capped with donor- and acceptor-substituted imidazole rings constitutes a diimidazole-derived push–pull, push–push, and pull–pull charge-transfer chromophore. The most common synthetic approach to diimidazoles, with the rings connected at C2, is shown in Scheme 1. Typical diimidazole chromophores in D-π-A arrangement (Figure 9) were investigated by Wu and Ye et al. [16–18,50–53]. Compounds **27a** (λ_max_ = 384 nm; β(AM1) = 50.91 × 10^−30^ esu; *T*_D_ = 377 °C) and **27c** (λ_max_ = 379 nm; β(AM1) = 29.5 × 10^−30^ esu; β(HRS) = 142 × 10^−30^ esu; *T*_D_ = 360 °C) with free amino or hydroxy groups were further used as reactive species for the functionalization of various polymers (see below).

**Figure 10:**
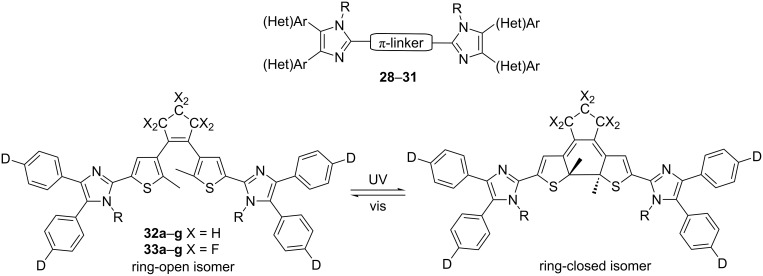
Typical D-π-D diimidazoles **28**–**31** [19,54–56] and photochromic diimidazoles **32**,**33** [57–58].

Within the last ten years, diimidazole D-π-D systems were extensively studied, in particular for their easy synthesis and unique properties [19,54–56]. Their general structure is shown in Figure 10 and selected properties are summarized in Table 6. Compounds **28**–**31** showed luminescent, photoluminescent, fluorescent or phosphorescent properties with the prospect for application in modern materials chemistry. This year, Liu, Yin, and co-workers [57–58] published a very nice example of photoswitchable diimidazole chromophores **32**,**33** with a distinct difference in optical properties between the open and closed forms.

**Table 6:** Structure and selected properties of diimidazoles **28**–**33** [19,54–58].

Comp.	π-linker/structure	(Het)Ar/D	R	λ_max,abs_ [nm]	λ_max,em_ [nm]	*T*_D_ [°C]	*Prospective application [reference]*

**28**	9,9,9’,9’,9’’,9’’-hexaoctylterfluorene	Ph	H	363^a^	433^b^	402	luminescence material [54]
**29**	poly-(1,4-phenylene)	4-C_8_H_17_Ph	H	378^c^	478^c^	220	photoluminescent material [19]
**30a**	1,4-phenylene	Ph	H	361^d^	424^d^	–	fluorescent materials – molecular photonics and sensing [55]
**30b**	1,4-phenylene	4-MeOPh	H	365^d^	440^d^	–	
**30c**	1,4-phenylene	Ph	Me	337^d^	417^d^	–	
**30d**	thiophen-2,5-diyl	Ph	H	385^d^	458^d^	–	
**30e**	thiophen-2,5-diyl	4-MeOPh	H	393^d^	470^d^	–	
**30f**	thiophen-2,5-diyl	Ph	Me	368^d^	452^d^	–	
**31a**	1,4-phenylene-thiophen-2,5-diyl	thiophene	H	385^e^	485^e^	479	fluorescent and phosphorescent materials – light-emitting device [56]
**31b**	2,2’-bithiophen-5,5’-diyl	thiophene	H	407^e^	513^e^	440	
**32a**	D = H; X = H	–	H	334/550^f^	400^g^	–	photochromic and fluorescent materials – optical switches [57]
**32b**	D = Me; X = H	–	H	336/552^f^	403^g^	–	
**32c**	D = OMe; X = H	–	H	340/554^f^	413^g^	–	
**32d**	D = NMe_2_; X = H	–	H	320/568^f^	462^g^	–	
**32e**	D = H; X = H	–	Me	296/542^f^	399^g^	–	
**32f**	D = Me; X = H	–	Me	296/542^f^	401^g^	–	
**32g**	D = OMe; X = H	–	Me	292/544^f^	412^g^	–	
**33a**	D = H; X = F	–	H	332/664^f^	–	–	photochromic materials – photoswitches and photoresponsive materials [58]
**33b**	D = Me; X = F	–	H	337/651^f^	–	–	
**33c**	D = OMe; X = F	–	H	343/682^f^	–	–	
**33d**	D = H; X = F	–	Me	318/630^f^	–	–	
**33e**	D = Me; X = F	–	Me	322/632^f^	–	–	
**33g**	D = OMe; X = F	–	Me	328/638^f^	–	–	

^a^Measured in 1,4-dioxane; ^b^measured in cyclohexane; ^c^measured in THF; ^d^measured in MeCN; ^e^measured in EtOH; ^f^absorption maxima of open-ring/closed-ring isomers measured in DMF; ^g^emission maxima of open-ring isomer (before UV irradiation) measured in DMF.

**Scheme 3:**
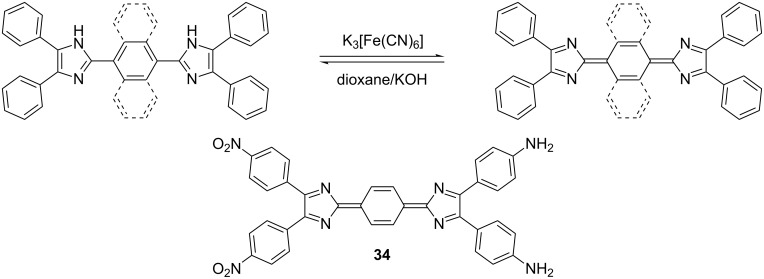
Oxidation of 1*H*-diimidazoles to 2*H*-diimidazoles (quinoids).

N-Unsubstituted diimidazoles can easily be oxidized to the corresponding quinoid structure (2*H*-imidazole derivatives), as shown in Scheme 3 [19,59–61]. In 1999, Ye et al. [61] reported the oxidation of D-π-A diimidazole **27a** to quinoid **34** and a comparison of the linear and nonlinear optical properties. Partially planarized quinoid **34** (μ *=* 19.0 D; β of 205.7 × 10^−30^ esu) showed a substantially higher dipole moment and first-order hyperpolarizability than chromophore **27a** (Figure 9; μ *=* 10.9 D; β = 50.91 × 10^−30^ esu) due to a higher efficiency of D-A conjugation.

### Benzimidazole-derived chromophores

**Figure 11:**
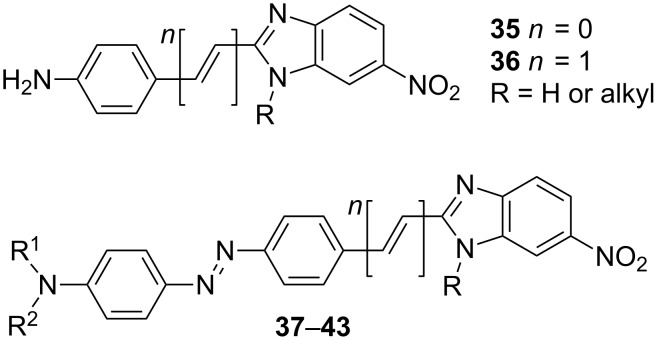
Typical benzimidazoles-derived D-π-A push–pull systems **35**–**43** [25,62–66].

In contrast to imidazoles, benzimidazoles possess fused benzene or higher (hetero)aromates, generally appended at C4/C5. This arrangement enables (i) an extension of the chromophore π-conjugated system; (ii) a planarization of the molecule; (iii) facile functionalization of the fused aromate by known methods; and (iv) a straightforward synthesis starting from inexpensive and readily available compounds (Scheme 1). Typical representatives of benzimidazole-derived D-π-A systems are shown in Figure 11. In 2004, Carella, Centore, and co-workers [25] reported the synthesis and further application of nitrobenzimidazole-derived anilines **35** and **36**. These two compounds were further used for the construction of various charge-transfer chromophores **37**–**43**, in particular by simple diazotation and subsequent azo-coupling of the terminal NH_2_ group [62–66]. Chromophores **37**–**43** found wide application as polymer dopants, cross-linkable organic glasses or inorganic–organic hybrid materials and showed high, stable, and tunable NLO performances, very good thermal stability, and, last but not least, easy synthesis from low-cost commercial precursors (Table 7).

**Table 7:** Structures and (N)LO properties of benzimidazoles **37**–**43** [25,62–64].

Comp.	*n*	R	R^1^	R^2^	λ_max,abs_^a^ [nm]	β*·*μ	*T*_D_ [°C]

**37**	0	H	CH_2_CH_2_OH	CH_2_CH_2_OH	472	940^b^	296
**38**	0	Et	CH_2_CH_2_OH	CH_2_CH_2_OH	466	950^b^	295
**39**	1	H	CH_2_CH_2_OH	CH_2_CH_2_OH	482	1550^b^	292
**40**	1	Et	CH_2_CH_2_OH	CH_2_CH_2_OH	487	1400^b^	314
**41**	0	Et	CH_2_CH_2_OMA^c^	CH_2_CH_2_OMA^c^	435	660^b^	300
**42**	0	H	CH_2_CH_2_OH	CH_2_CH_3_	476	2306^d^	274
**43**	1	H	CH_2_CH_2_OH	CH_2_CH_3_	480	3129^d^	244

^a^Measured in DMF; ^b^measured in DMF by EFISH technique at 1907 nm (10^−48^ esu); ^c^MA = methacrylate; ^d^measured by solvatochromic method at 1907 nm (10^−30^ esu·D).

**Figure 12:**
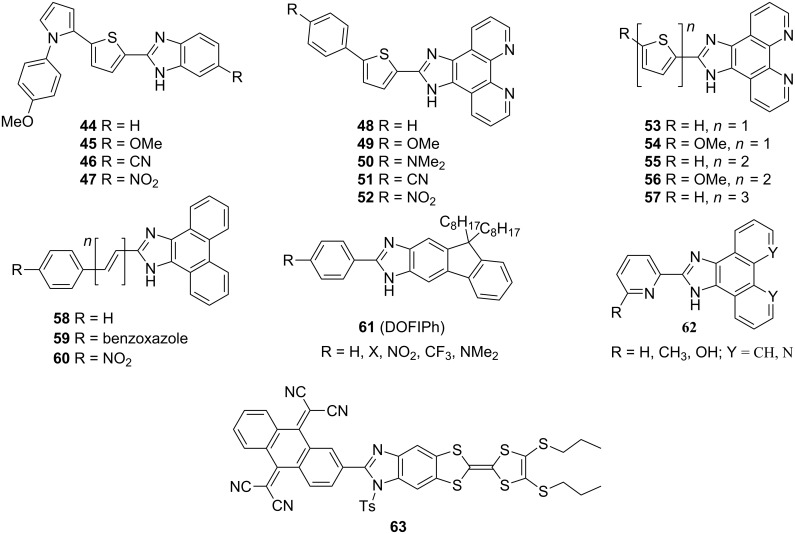
Structure of benzimidazoles (**44**–**47**), imidazophenanthrolines (**48**–**57**), imidazophenanthrenes (**58**–**60**), fluorophores **61**, **62**, and TCAQ-imidazo-TTF (**63**) chromophores [23–24,67–71].

Raposo and co-workers investigated benzimidazole derivatives **44**–**47** with either a donor- or acceptor-substituted benzene ring, whose π-conjugated pathways comprise thiophene and pyrrole subunits [24]. This series of chromophores was further extended by arylthienylimidazole phenanthrolines **48**–**52** and oligothienylimidazole phenanthrolines **53**–**57** (Figure 12; [23,67]). The benzo[*d*]imidazole core in compounds **46**–**57** behaves as an electron acceptor and, when substituted with electron donors at C2, an efficient ICT can be achieved. Consequently, the measured hyperpolarizabilities β increase with the rise in donating ability of the appended donors or extension of the π-conjugated path. Thiophene, used as a part of the π-linker, particularly in chromophores **53**–**57**, caused β enhancement up to 320 × 10^−30^ esu (Table 8). This clearly demonstrates the beneficial role of the thiophene as a polarizable unit and auxiliary electron donor. A combination of fused phenanthroline-imidazole acceptor moiety, *N*,*N*-dimethylamino donor, and arylthienyl π-linker, as in **50**, resulted in a CT chromophore with β = 189 × 10^−30^ esu. It should also be noted that all chromophores showed exceptionally high thermal stability with *T*_D_ up to 470 °C.

**Table 8:** Structure and properties of chromophore **44**–**57** [23–24,67].

Comp.	*n*	R	λ_max_^a^ [nm]	β^b^ [10^−30^ esu]	*T*_D_ [°C]

**44**	–	H	361	60	380
**45**	–	OMe	364	–	401
**46**	–	CN	367	114	390
**47**	–	NO_2_	363	121	365
**48**	–	H	361	41	470
**49**	–	OMe	370	145	431
**50**	–	NMe_2_	391	189	448
**51**	–	CN	386	91	465
**52**	–	NO_2_	408	45	450
**53**	1	H	337	26	441
**54**	1	OMe	346	110	341
**55**	2	H	384	46	451
**56**	2	OMe	393	170	423
**57**	3	H	412	320	467

^a^Measured in 1,4-dioxane; ^b^first-order hyperpolarizability measured in 1,4-dioxane by hyper-Rayleigh scattering (HRS) method at 1064 nm.

Recently, Cui et al. investigated simple phenanthro[9,10-*d*]imidazoles **58**–**60** as two-photon absorbing molecules with blue upconversion fluorescence [68]. These imidazole derivatives proved to be potent two-photon absorbing molecules with TPA cross-section δ up to 20.65 GM at 800 nm. The molecular structure of chromophore **60** was also confirmed by X-ray analysis. Similar derivatives **61** (DOFIPh), based on the fluoreno[2,3-*d*]imidazole core, showed strong and tunable blue emission in the solid state (λ_max,em_ = 417–526 nm in film), which makes these molecules potentially applicable as active layers for OLEDs [69]. Chromophores **62** were investigated as photoluminescence materials with λ_max,abs_ = 324–367 nm and λ_max,em_ = 393–470 nm, respectively [70].

In 2007, Liu et al. reported a very nice example of D-π-A system **63** based on benzimidazole as a parent π-conjugated backbone fused with TCAQ (tetracyanoanthraquinodimethane) and TTF (tetrathiafulvalene) as acceptor and donor moieties, respectively [71]. This molecule was investigated in terms of absorption spectroscopy, X-ray analysis, and electrochemistry and showed remarkable responses as a function of pH. Unfortunately, no NLO properties were investigated.

**Scheme 4:**
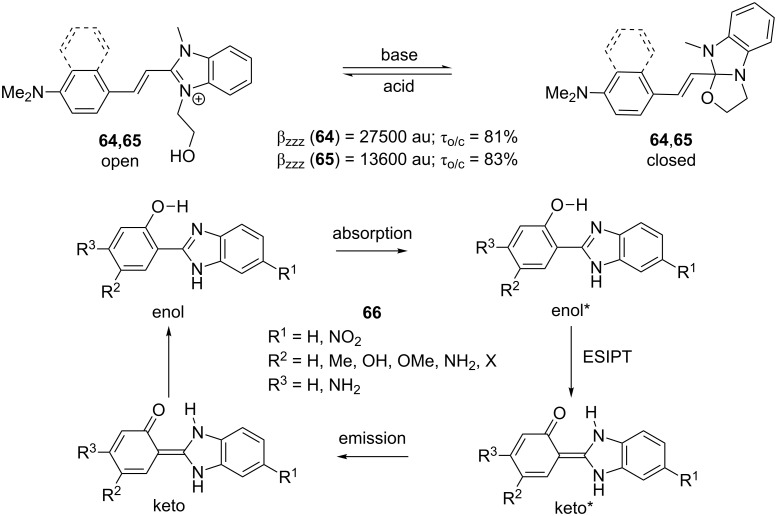
Acidoswitchable NLO-phores **64**,**65** and ESIPT mechanism [72–74].

Benzimidazole-derived compounds were recently also used as chromophores with switchable properties. Benzimidazolo[2,3-*b*]oxazolidines **64**, **65** showed acidochromic behavior with remarkable contrast τ_o/c_ in the NLO responses along the reversible transformation observed by HRS (Scheme 4; [72]). Whereas the open form of **64**, **65** with strong ICT showed λ_max_ at 402 and 406 nm and longitudinal hyperpolarizabilities β_zzz_ at 27500 and 13600 au, the closed form showed only diminished nonlinearities due to the interruption of efficient D-A conjugation.

Compounds showing excited-state intramolecular proton transfer (ESIPT) represent another example of switchable NLO-phores (Scheme 4). Donor- and acceptor-substituted push–pull systems **66** based on 2-(2-hydroxyphenyl)benzo[*d*]imidazole showed efficient photoinduced blue-green proton-transfer fluorescence [73–74]. Taking the amino/nitro-substituted derivative as an example (R^1^ = NO_2_; R^2^ = H; R^3^ = NH_2_; LEN [73]), this compound showed absorption and emission maxima at 373 and 448 nm, respectively, and large first-order hyperpolarizability β = 1197 × 10^−30^ esu. The combination of such properties makes this compound a promising material for storing information at the molecular level.

**Figure 13:**
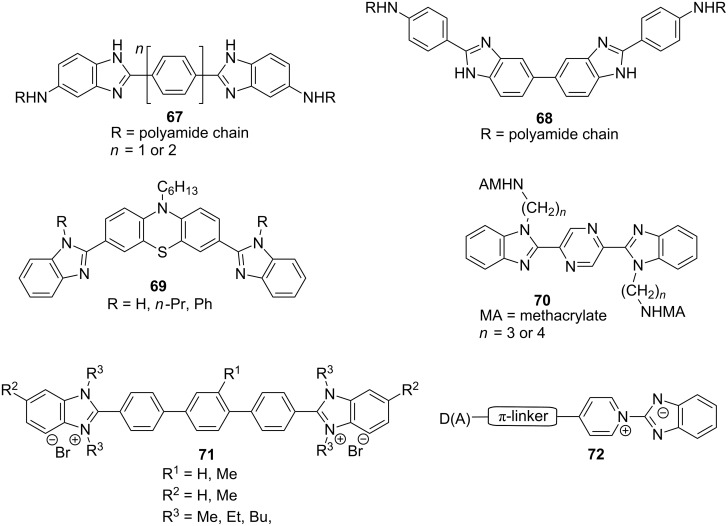
General structures of bis(benzimidazole) chromophores **67**–**71** and pyridinium betaines **72** [75–79].

Similar to diimidazole compounds **27**–**34**, two benzimidazole cores may also be incorporated into the chromophore backbone. The molecular structures of recently investigated bis(benzimidazole)-derived chromophores **67**–**71** are shown in Figure 13. All these bis(benzimidazole) systems were primarily studied as fluorescent compounds. Polymeric chromophores **67** and **68** showed blue fluorescence with emission maxima at 410–515 nm [75]. A-π-D-π-A molecules **69** featuring a central phenothiazine donor moiety and two peripheral benzimidazole acceptor units were investigated by Ahn et al. [76]. These ambipolar molecules possess energy levels that are well-matched with the Fermi levels of the electrodes to facilitate the electron or hole injection and transfer in OLED devices. 2,5-Bis(benzimidazol-2-yl)pyrazine derivatives **70** (BBIP), with improved solubility through *N*,*N’*-dialkylation, exhibited high fluorescence intensity even in protic solvents, as well as interesting solvatochromic properties [77]. Terphenyl-bridged bis(benzimidazolium) salts **71**, soluble in water and common organic solvents, emit blue light with λ_max,em_ at 420–441 nm in thin films [78]. This feature makes them potentially applicable as blue-light emitters in OLEDs.

Benzimidazole-based push–pull systems were studied also theoretically. Abe et al. studied pyridinium betaines of general formula **72** consisting of negatively charged benzimidazolate and a positively charged pyridinium ion (Figure 13; [79]). Moreover, the π-conjugated system was systematically enlarged and either donor- or acceptor-substituted in order to generate D-π-A-π-D and D-π-A-π-A systems. The performed ab initio and INDO/S MO calculations of ground-state dipole moments and first-order hyperpolarizabilities β revealed that the latter chromophore arrangement resulted in significantly enhanced nonlinearities. The benzimidazolate anion as a donor moiety was quantum-chemically studied also by Xu, Su, and co-workers [80]. Structurally highly similar chromophores to **44**–**47** (Figure 12), reported by Raposo [24], were investigated by means of molecular geometry optimization, absorption/emission spectra, first-order hyperpolarizability calculations, and simulation of NH proton abstraction by using a fluoride anion. Remarkably large differences between the β values of protonated/deprotonated forms showed that benzimidazoles are potent molecules for a new type of NLO molecular switching.

### Chromophores featuring a 4,5-dicyanoimidazole acceptor moiety

**Figure 14:**
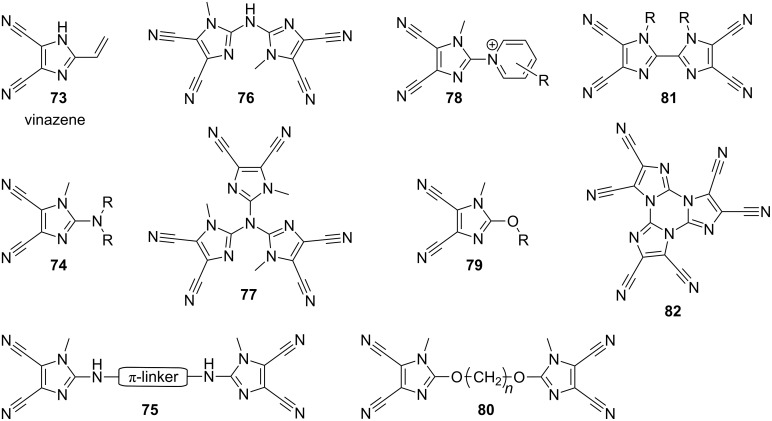
Overview of 4,5-dicyanoimidazole derivatives investigated by Rasmussen et al. [29,81–94].

Since the discovery of 4,5-dicyanoimidazole by Woodward in 1950 (Scheme 2; [27]), this imidazole derivative has become one of the “standard acceptor moieties” used in materials organic chemistry. The primary development and popularization of this molecule can be ascribed to Rasmussen and co-workers as early as the 1980s–1990s. Over a period of 20 years, Rasmussen et al. published an admirable number of articles dealing with the synthesis, combination, functionalization, and application of 4,5-dicyanoimidazoles. Figure 14 shows a selection of Rasmussen’s 4,5-dicyanoimidazole derivatives, such as vinazene **73** [29,81], push–pull amines and betaines **74**–**78** [82–87], alkoxy derivatives **79**,**80** [88], biimidazoles **81** [89–92], and triimidazoles **82** [93–94], as well as fullerenes [95] and polymers [96–99].

The chemistry of 4,5-dicyanoimidazole was reviewed in 1987 by Donald and Webster [100] and its application in liquid-crystal media and devices was again summarized in a Merck patent in 2004 [101].

**Figure 15:**
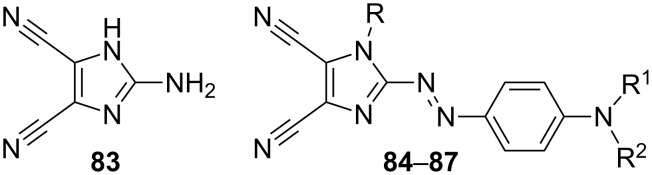
4,5-Dicyanoimidazole-derived chromophores **84**–**87** [103–106].

In 2004 and 2005, Carella, Centore, and co-workers utilized 2-amino-4,5-dicyanoimidazole **83** (for X-ray structure analysis, see [102]) in the synthesis of chromophores **84**–**86** featuring central phenylazo π-linker, 4,5-dicyanoimidazole as acceptor, and *N*,*N*-dialkylamino donor (Figure 15; [103–104]). The nonlinear optical properties of these three chromophores were investigated by EFISH experiment (Table 9). The molecular structure of chromophore **84** was also confirmed by X-ray analysis. These chromophores, with free terminal OH-functions, were further used as monomers for copolymerization with polyester, polyuretane, and polymethacrylate (see below). Structurally very similar chromophore **87** (R = H; R^1^ = CH_2_CH_2_OH; R^2^ = Et) was used for incorporation into the sol–gel hybrid films based on alkoxysilanes [105–106]. This new material is to be applied as an electro-optic modulator.

**Table 9:** Structures, optical (linear and nonlinear), and thermal properties of chromophores **84**–**86** [103–104].

Comp.	R	R^1^	R^2^	λ_max_^a^ [nm]	μ·β^b^ [10^−48^ esu]	*T*_D_ [°C]

**84**	H	CH_2_CH_2_OH	CH_2_CH_2_OH	462	1050	230^c^
**85**	H	CH_3_	CH_2_CH_2_OMA	459	1000	249
**86**	Et	CH_3_	CH_2_CH_2_OMA	496	800	236

^a^Measured in DMF (**84**) and CHCl_3_ (**85**, **86**); ^b^measured in DMF by the EFISH technique at 1907 nm; ^c^melting point.

**Figure 16:**
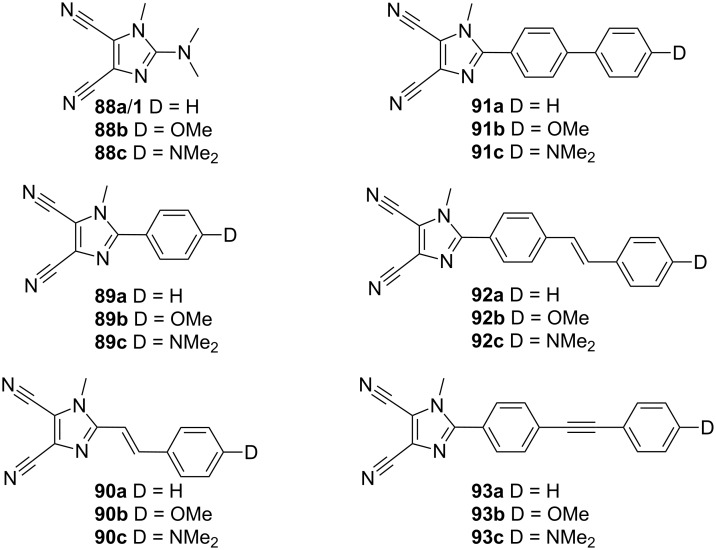
Push–pull chromophores **88**–**93** with systematically extended π-linker [30].

Our synthetic efforts in the field of 4,5-dicyanoimidazole-derived chromophores began with the initial set of push–pull molecules **88**–**93** (Figure 16; [30]). Chromophores **88**–**93** were synthesized by Suzuki–Miyaura cross-coupling reactions [107] on 2-bromoimidazole **2** (Scheme 2) as three series **a**, **b**, and **c** according to the type of the used donor D (H, OMe, and NMe_2_). The π-conjugated path was systematically varied and enlarged in order to study its influence on the chromophore polarizability. The chromophores were primarily investigated by electronic-absorption spectra, electrochemistry, X-ray analysis, and quantum-chemical calculations. The resulting data set was further processed by factor analysis to deduce the structure–property relationships. The most important structural factors affecting the (non)linear optical properties and electrochemical behavior are (i) the presence of a strongly conjugating donor and (ii) the length and (iii) planarity of the π-conjugated system. In this respect, chromophores **90c**, **92c**, and **93c** seem to possess one of the better balances between performance and practicality within the studied series.

The photoinduced absorption, birefringence, and second-harmonic generation of chromophores **88c**–**93c** (D = NMe_2_) embedded within polymethylmethacrylate matrices were studied and complimented by quantum-chemical calculations. These doped polymer films showed very efficient and tunable nonlinearities with β_av_ ranging from 899 to 25798 au (Table 10; [108]).

**Table 10:** Properties of chromophores **88**–**93** [30,108–110].

Comp.	D	Δ(*E*_ox,1_−*E*_red,1_)^a^ [V]	*E*_HOMO_−*E*_LUMO_ [eV]	λ_max,abs_^b^ [nm (eV)]	λ_max,em_^c^ [nm]/Φ	β^d^ [10^−30^ esu]	β_av_^e^ [au]	β_zzz_^f^ [au]

**88a**	H	–	9.19	244 (5.08)	–	1.5	–	–
**88b**	OMe	4.09	8.63	271 (4.58)	–	3.5	–	–
**88c**	NMe_2_	3.34	8.51	293 (4.23)	361/0.05	2.7	899	–
**89a**	H	4.06	8.69	264 (4.70)	320/0.28	2.6	–	–
**89b**	OMe	3.65	8.30	275 (4.51)	354/0.65	8.3	–	–
**89c**	NMe_2_	2.85	7.73	316 (3.92)	452/0.37	14.6	5657	9710
**90a**	H	3.37	7.98	313 (3.96)	–	5.3	–	–
**90b**	OMe	3.08	7.69	331 (3.75)	–	18.2	–	–
**90c**	NMe_2_	2.50	7.27	381 (3.25)	470/0.04	32.7	16750	19708
**91a**	H	3.70	8.38	286 (4.34)	351/0.87	5.2	–	–
**91b**	OMe	3.34	7.90	301 (4.12)	388/0.98	13.0	–	–
**91c**	NMe_2_	2.64	7.31	346 (3.58)	485/0.64	21.9	10754	14408
**92a**	H	3.30	7.78	325 (3.82)	390/0.59	13.2	–	–
**92b**	OMe	3.03	7.47	331 (3.75)	425/0.15	30.7	–	–
**92c**	NMe_2_	2.39	7.07	380 (3.26)	528/0.53	49.1	25978	18660
**93a**	H	3.63	7.96	308 (4.03)	361/0.80	9.3	–	–
**93b**	OMe	3.25	7.63	323 (3.84)	396/0.83	22.8	–	–
**93c**	NMe_2_	2.50	7.17	364 (3.41)	515/0.73	37.1	23401	24674

^a^Measured by DC polarography and RDV, potentials are given vs. SCE; ^b^absorption maxima measured in CH_2_Cl_2_; ^c^emission maxima/quantum yields measured in EtOAc; ^d^PM3/PM6 calculated values (MOPAC); ^e^measured in poly(methyl methacrylate) by SHG experiment at 1064 nm; ^f^longitudinal molecular first hyperpolarizabilities measured in CH_2_Cl_2_ by HRS experiment at 1064 nm.

**Figure 17:**
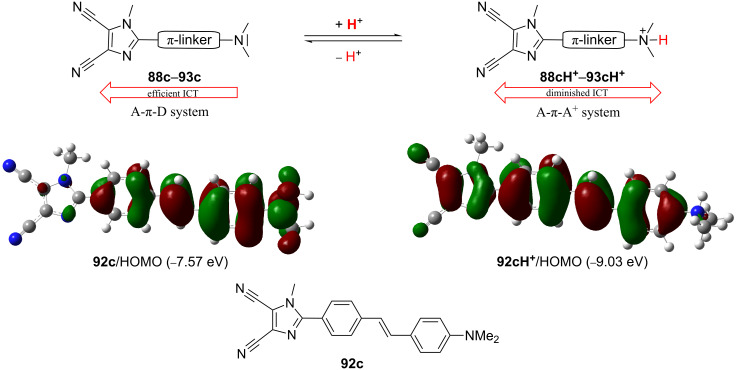
pH-triggered NLO switches **88c**–**93c** [109].

Moreover, the *N*,*N*-dimethylamino donor in **88c**–**93c** can easily be protonated. Whereas in the unprotonated form (**88c**–**93c**), an efficient ICT from the donor to the acceptor exists (D-π-A system), in the protonated forms (**88cH^+^**–**93cH^+^**) only diminished ICT between the π-linker and the peripheral acceptors A and A^+^ takes place (Figure 17; [109]). This results in a high contrast in the nonlinearities between both forms (Table 11) as well as in a raised energy and character of the HOMO (Figure 17). Hence, chromophores **88c**–**93c** proved to be very efficient pH-triggered NLO switches.

**Table 11:** HRS first hyperpolarizabilities (β) and depolarization ratios (DR) of **88c**–**93c** before/after protonation (CH_2_Cl_2_) [109].

Comp.	Unprotonated		Protonated		Contrast
	β_HRS_ (−2ω;ω;ω) [au]	DR	β_HRS_ (−2ω;ω;ω) [au]	DR	

**88c**	379	4.87	114	1.78	3.32
**89c**	1938	5.48	256	1.65	7.57
**90c**	10485	5.11	541	1.87	19.38
**91c**	3264	5.40	290	2.28	11.26
**92c**	8485	5.15	361	1.78	23.50
**93c**	8236	5.15	639	2.44	12.89

The fluorescent and photophysical properties of chromophores **88**–**93** were further studied [110–111]. The fluorescence was studied in various solvents and polymer matrices and at various temperatures. Intense fluorescence with quantum yields of 0.05 to 0.98 was observed in nonpolar solvents and polymer matrices within the range of 320 to 528 nm (Table 10).

**Figure 18:**
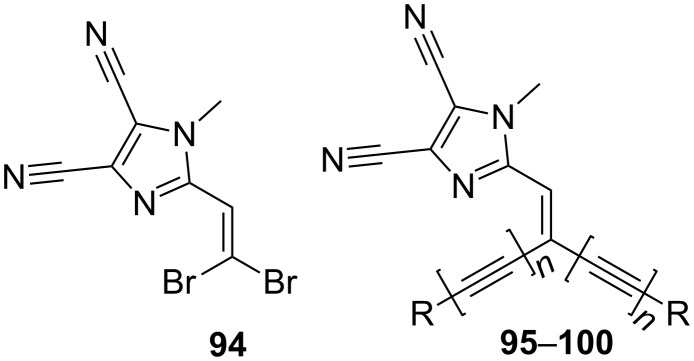
Dibromoolefin **94** and branched chromophores **95**–**100** [112–113].

The first set of 4,5-dicyanoimidazole-derived chromophores **88**–**93** possessed only one donor at the imidazole C2. Hence, our further synthetic efforts were focused on the synthesis of branched chromophores **95**–**100** (Figure 18; [112]). The synthesis of this series of chromophores involved two-fold Suzuki–Miyaura and Sonogashira cross-coupling reactions on dibromoolefin **94** (for X-ray structure see [113]). This compound proved to be a very useful, fully planar precursor for the construction of a chromophore π-conjugated backbone. In contrast to **88**–**93**, the presence of two (or four) *N*,*N*-dimethylamino donors and the systematic extension of the π-linkers in **95**–**100** resulted in a bathochromically shifted CT-band, lowered electrochemically measured and calculated HOMO–LUMO gaps, and enhanced first-order hyperpolarizability up to 70 × 10^−30^ esu (Table 12).

**Table 12:** Structures and selected properties of branched chromophores [112].

Comp.	R	*n*	Δ(*E*_ox,1_−*E*_red,1_) [V]	*E*_HOMO_−*E*_LUMO_ [eV]	λ_max_^a^ [nm (eV)]	β^b^ [10^−30^ esu]

**95**	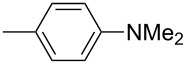	0	2.80	7.48	349 (3.55)	18.3
**96**	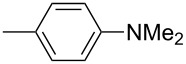	1	2.35	6.85	429 (2.90)	31.2
**97**	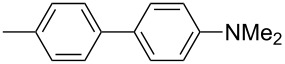	1	2.10	6.68	416 (2.98)	33.1
**98**	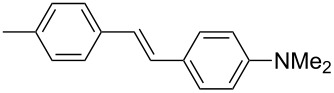	1	1.84	6.48	437 (2.84)	70.2
**99**	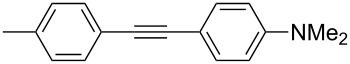	1	2.10	6.61	407 (3.05)	49.0
**100**	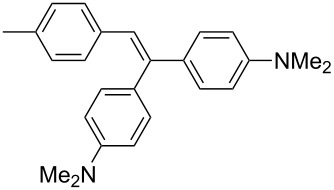	1	2.00	6.64	450 (2.76)	32.6

^a^Measured in CH_2_Cl_2_; ^b^average second-order polarizabilities calculated by PM3/PM6 methods (MOPAC).

**Figure 19:**
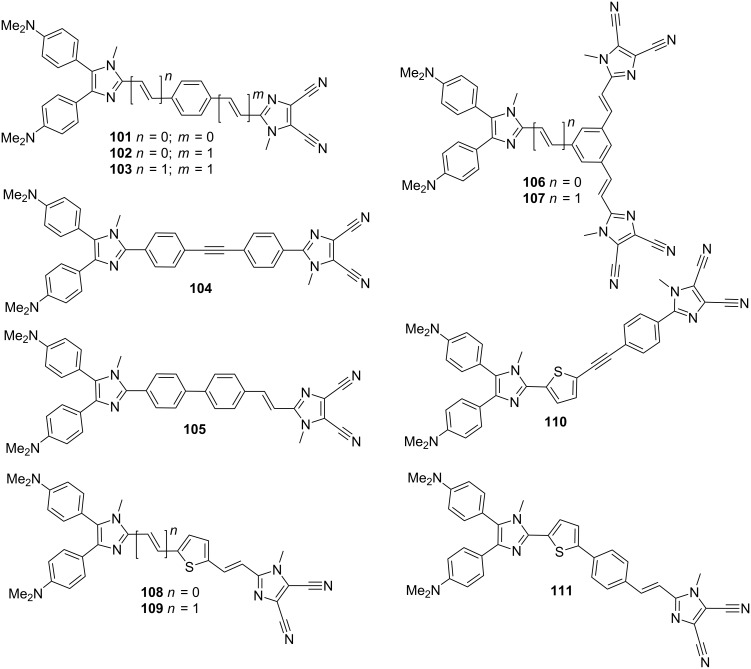
Imidazole as a donor–acceptor unit in CT-chromophores **101**–**111** [20].

**Figure 20:**
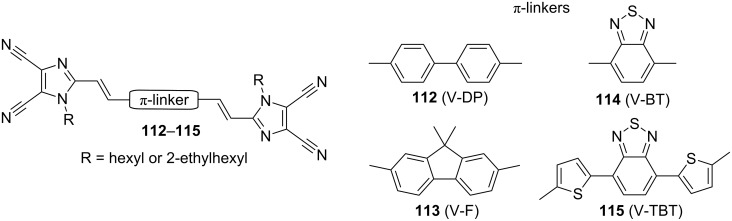
Diimidazoles **112**–**115** used as small electron acceptors in organic solar cells [115–116].

A combination of donor and acceptor 4,5-disubstituted imidazoles, namely 4,5-bis[4-(*N*,*N*-dimethylamino)phenyl]imidazole and 4,5-dicyanoimidazole as in **21**–**26** (Figure 8) and **88**–**100** (Figure 16 and Figure 18), respectively, resulted in diimidazole-type chromophores **101**–**111** (Figure 19; [20]). In contrast to a typical synthetic approach to diimidazoles as shown in Scheme 1, we used 4,5-dicyanoimidazole derivatives **1**–**3** (Scheme 2) and modern direct arylation, Suzuki–Miyaura, Sonogashira, and Heck reactions to construct molecules **101**–**111**. These chromophores possess two (or three) imidazole parent π-backbones, either as donor or acceptor moieties, and a systematically extended π-linker. Thiophene, in combination with double bonds, was used as a highly polarizable subunit of the π-linker, which resulted in very efficient chromophores with first- and second-order hyperpolarizabilities β and γ up to 526 × 10^−30^ and 315 × 10^−27^ esu, respectively (Table 13, chromophore **109**). In general, this series of diimidazole-based compounds featured the most efficient NLO-phores.

**Table 13:** Diimidazole chromophores **101**–**111**; properties [20].

Comp.	Δ(*E*_ox,1_−*E*_red,1_) [V]	*E*_HOMO_−*E*_LUMO_ [eV]	λ_max_^a^ [nm (eV)]	β^b^ [10^−30^ esu]	γ^b^ [10^−27^ esu]

**101**	2.39	6.63	366 (3.39)	38.2	3.61
**102**	2.17	6.48	404 (3.07)	38.2	5.17
**103**	2.08	6.35	444 (2.79)	66.0	8.99
**104**	2.31	6.35	373 (3.32)	44.8	6.26
**105**	2.17	6.38	382 (3.25)	38.4	5.78
**106**	2.12	6.49	316 (3.92)	25.5	4.51
**107**	2.10	6.44	394 (3.15)	39.6	5.97
**108**	2.11	6.65	448 (2.77)	299.0	164.05
**109**	1.95	6.14	479 (2.59)	526.3	315.15
**110**	2.27	6.80	413 (3.00)	82.2	45.91
**111**	2.11	6.47	420 (2.95)	47.9	20.18

^a^Measured in CH_2_Cl_2_; ^b^average second/third-order polarizabilities calculated by PM3/PM6 methods (MOPAC).

Organic π-conjugated materials based on 4,5-dicyanoimidazole were recently developed as opto-electronic materials with a practical application. For instance, in 2002 Yang et al. [114] reported a fairly simple organic-electrical bistable device (OBD) based on amine **83** (Figure 15). Yang’s OBD consisted of organic material based on **83** with a built-in thin aluminum active layer. The OBD’s conductivity in the two electric states was considerably different, and, moreover, the OBD showed remarkable stability without significant device degradation over a million write–erase cycles. Hence, the performance of this device makes OBD attractive for application in rewritable memory cells. In 2007, Sellinger et al. became very interested in the Heck coupling of *N*-alkyl vinazenes with various (hetero)aromates [115]. This synthetic interest resulted in four new diimidazole compounds **112**–**115** (Figure 20). This series of basic π-conjugated compounds was significantly extended in 2009 by a library of various π-linkers [116]. As a materials researcher, Sellinger applied these *n*-type conjugated materials as small-molecule electron acceptors. The combination of V-BT (**114**) with polyhexylthiophene donor (P3HT) in an initial organic solar cell showed high external quantum efficiencies exceeding 14%. Sellinger’s further efforts were focused on improving optical, photovoltaic, and charge-transport properties as well as efficiencies of V-BT derived solar cells. Thus, he studied new processing techniques for solar cells, the use of various semiconducting donor polymers, nanoimprint lithography, etc. [117–120]. This effort resulted in organic photovoltaic devices with a very high fill factor FF = 57% and an external quantum efficiency IPCE (incident photons converted to electrons) = 27%. These values rival those measured for popular fullerene acceptors.

### Imidazole chromophores incorporated into the polymer

Recently, imidazole-derived CT chromophores found wide application either as polymer dopants (guest–host systems) or in polymers with chemically bonded NLO-phores (side-chain, main-chain, and cross-linked). An incorporation of the chromophore into the polymer backbone brings with it a higher and facile polarizability, higher thermal stability, and NLO responses as well as prospective applicability in modern materials chemistry. The second-order susceptibilities of nonlinear optical polymers are historically referred to as “*d*_ij_” coefficients (1/2 of the respective χ_ij_^(2)^ values). The electro-optic coefficient *r*_ij_, indicating the degree of the refractive index change caused by a unit increase in the voltage applied across the polymer film, is another important feature of the nonlinear optical polymer waveguides. The relationship between the *d* and *r* coefficients can be simplified according to the following equation

[1]
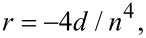


where *n* is the index of refraction. However, only two components of the *d* and *r* coefficients that are parallel and perpendicular to the average dipolar chromophore axis are important and investigated (*d*_33_, *d*_31_ and *r*_33_, *r*_31_). The physical stability of the nonlinear optical polymers refers to the stability of alignment of the chromophore. The glass transition temperature (*T*_g_) and the decomposition temperature (*T*_D_) are the most widely provided parameters of polymer physical stability. The polar order of the polymer (centrosymmetry removal) is usually achieved by the electric-field, thermal (*T*_p_) and optical poling procedures [121]. Only the polymer systems with covalently attached imidazole CT chromophores will be discussed in the following section.

**Figure 21:**
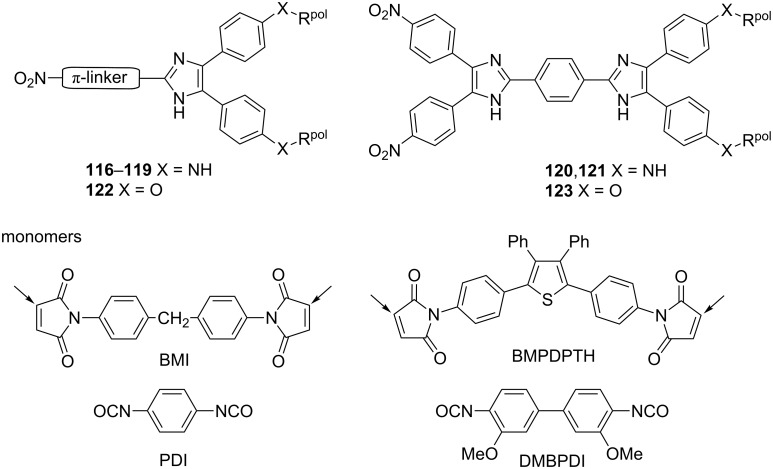
Amino- and hydroxy-functionalized chromophores incorporated into a polymer backbone R^pol^ [18,50–53,122–124].

4,5-Bis(4-aminophenyl)(bi)imidazole (e.g. **15**/**27a**; Figure 6/Figure 9) and 4,5-bis(4-hydroxyphenyl)diimidazole (i.e., **27c**; Figure 9) represent simple chromophores with free NH_2_ and OH peripheral groups, which can be used to link the chromophore to various polymers (Figure 21). These systems were mainly investigated by Ye et al. (Table 14; [18,50–53,122–124]). The polyimides **116**–**118** (X = NH) were prepared by the copolymerization (Michael addition) of *N*,*N*’-bismaleiimido-4,4’-diphenylmethane (BMI) with Y-shaped imidazole chromophores **15** featuring a slightly extended π-linker. These polymers were thermally poled to achieve moderate nonlinearity and good thermal stability [122–123]. Similar reaction of 2,5-bis(4-*N*-maleiimido)phenyl-3,4-diphenylthiophene (BMPDPTH) with chromophore **15f** afforded system **119** with significantly enhanced nonlinearity (*d*_33_ = 32.2 pm/V) [124] as a result of the π-linker extension through the thiophene and double-bond subunits. Diimidazole **27a** (X = NH) was also utilized as a reactive chromophore for copolymerization with BMPDPTH and 1,4-phenylene diisocyanate (PDI) to provide polyimide **120** and polyurea **121** [50–53]. Ye also investigated the similar (bi)imidazole-derived polymers **122** and **123** (X = OH) with a polyuretane backbone generated after copolymerization with 3,3’-dimethoxy-4,4’-biphenylene diisocyanate (DMBPDI) [18]. However, the measured nonlinearities and thermal stabilities of these polymers did not exceed that measured for **119** (Table 14).

**Table 14:** Nonlinear optical polymers **116**–**123**; properties [18,50–53,122–124].

Comp.	Chromophore/π-linker	X	Monomer	*d*_33_ [pm/V]	*T*_g_ [°C]	*T*_D_ [°C]

**116**	**15a** (65%)/–(C_6_H_4_)–	NH	BMI	–	262	335
**117**	–N=N–(C_6_H_4_)–	NH	BMI	13.6	250	331
**118**	–CH=CH–(C_6_H_4_)–	NH	BMI	11.3	258	335
**119**	**15f**/–(C_4_H_2_S)–CH=CH–(C_6_H_4_)–	NH	BMPDPTH	32.2	304	330
**120**	**27a**	NH	BMPDPTH	16.4	234	380
**121**	**27a**	NH	PDI	24.0	272	290
**122**	–(C_6_H_4_)–	O	DMBPDI	12.0	202	300
**123**	**27c**	O	DMBPDI	15.0	223	335

**Figure 22:**
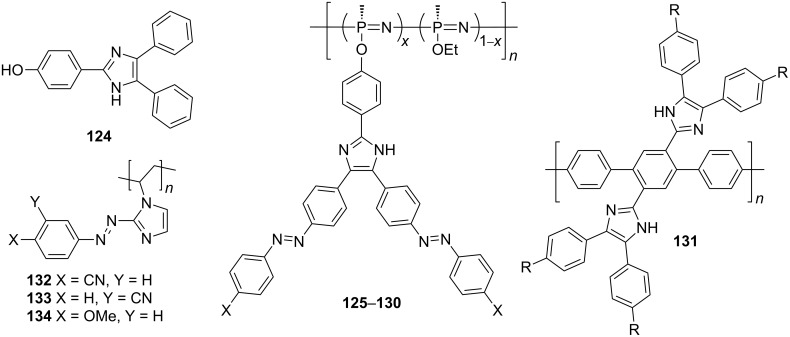
Structure of polyphosphazene polymers bearing NLO-phores [125–127] and some other recent examples of nonlinear optical polymers [19,128].

Tang et al. showed another approach to producing nonlinear optical polymers. The synthetically easily available hydroxy lophine **124** was covalently bonded to the polyphosphazene backbone and subsequently modified by post-azo coupling with variously substituted benzenediazonium salts to afford systems **125**–**130** (Figure 22; Table 15; [125–127]). These systems possess good optical transparency, high *T*_g_, and large *d*_33_ (SHG) and photoinduced birefringence values relative to those known for polyphosphazenes to date. Last but not least, this simple synthetic pathway opens space for manifold elaboration and functionalization of various prepolymers in order to enhance their nonlinearities.

**Table 15:** Properties of polyphosphazenes **125**–**130** [125–127].

Comp.	X	λ_max_ [nm]	*d*_33_ [pm/V]	*T*_g_ [°C]	Δ*n*^a^ [10^−2^]

**125**	NO_2_	363	–	170	0.45
**126**	Cl	363	29	158	–
**127**	F	372	37	157	1.32
**128**	I	365	23	169	–
**129**	Me	354	–	165	1.01
**130**	OMe	375	–	174	1.12

^a^Photoinduced birefringence measured at 633 nm (He–Ne laser).

Recently, Müllen et al. [19] as well as Koszykowska et al. [128] contributed to the field of nonlinear optical polymers (Figure 22). Müllen’s imidazole-functionalized poly(*p*-phenylene) **131** proved to be a promising hole-transporting emissive material, which can be oxidized to quinoid (Scheme 3) with an additional low-wavelength absorption at 655 nm (light-absorbing material for solar cells). In 2009, Koszykowska et al. demonstrated facile polymerization of 1-vinylimidazole and subsequent post-azo coupling at imidazole C2 to attach various donor- and acceptor-substituted pendants. Moreover, the poly(*N*-vinyl-2-(phenylazo)imidazoles **132**–**134** showed interesting switchable photochromic properties.

Typical representatives of benzimidazole CT chromophores **37**–**43**, intended as reactive monomers for incorporation into the polymer backbone, were investigated by Carella, Centore et al. [62–63] and Cross et al. [65–66] and are shown in Figure 11. The chromophores **37**–**43** were attached to polyuretane and polyester by solution copolymerization with tolylene-2,4-diisocyanate (TDI), (2-methoxy)terephthaloyl dichloride [(M)TPC], and isophthaloyl dichloride (IPC) to afford nonlinear optical polymers **135**–**140**. Polymers **141**–**143** were synthesized by AIBN-promoted polymerization of the methacrylate terminal functionality. Table 16 summarizes the structures, SHG coefficients *d*_33_, and stability parameters *T*_g_ and *T*_D_. It is obvious that the three cross-linked nonlinear optical polymers **141**–**143**, prepared by radical polymerization, possess much higher nonlinearities than the two-component polymers **135**–**140**. However, the achieved nonlinearities are still lower than those measured for previous polymeric systems, e.g., **119** and **126**–**128**.

**Table 16:** Benzimidazole-derived chromophores embedded into a polymer **135**–**143** [62–63,65].

Comp.	Chromophore/ Structure^a^	Monomer	*d*_33_ [pm/V]	*T*_g_ [°C]	*T*_D_ [°C]

**135**	**38**	TDI	1.8	158	275
**136**	**40**	TDI	1.2	171	292
**137**	**38**	TPC	2.0	149	311
**138**	**38**	MTPC	2.2	146	327
**139**	**40**	MTPC	0.9	173	292
**140**	**38**	IPC	2.3	147	313
**141**	**41**		14.0	37	300
**142**	R = CH_2_CH(CH_3_)OH R^1^ = CH_2_CH_2_OC(O)NHCH_2_CH_2_OMA R^2^ = CH_2_CH_3_		13.0	128	–
**143**	R = CH_2_CH(CH_3_)OH R^1^ = CH_2_CH_2_OMA R^2^ = CH_2_CH_3_		16.5	151	–

^a^See Figure 11.

Chromophores **67** and **68** attached to polyamide and polyester backbones by copolymerization with *m*-phenylenediamide (MPD) and isophthaloyl dichloride (IPC) as well as bis(benzimidazolyl)pyrazines **70** (Figure 13; [75,77]) represent further examples of interesting polymers functionalized with benzimidazole-based CT chromophores. Unfortunately, no NLO properties were investigated. In 2002, Kudryavtsev et al. reported third-harmonic generation in copolymer films (polyamides) featuring a *N*-phenylbenzimidazole motif [129]. These materials exhibited their longest absorption maxima λ_max_ at 490–515 and third-order NLO susceptibility χ^(3)^(3ω;ω,ω,ω) within the range of 1.5 to 2.6 × 10^−13^ esu (measured by THG at 1064 nm).

**Figure 23:**
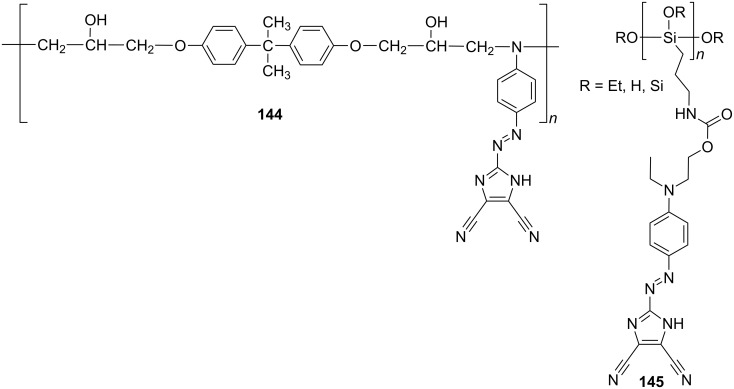
Epoxy- and silica-based polymers functionalized with 4,5-dicyanoimidazole unit [105,130].

Variously 4,5-dicyanoimidazole-functionalized polymers were mainly investigated by Rasmussen et al. [29,81,86–88,96–99]. However, these systems were not intended as nonlinear optical polymers. Their properties were primarily studied by electrochemistry, absorption spectroscopy, NMR, FTIR spectroscopy, DSC, and TGA. Nevertheless, in 1998, Tripathy and co-workers reported the synthesis of epoxy-based nonlinear optical polymers **144** functionalized by post-azo coupling (Figure 23; [130]). The parent polymer backbone was synthesized from diglycidyl ether of bisphenol A and aniline and was further functionalized by diazotized amine **83** (Figure 15). This polymeric material possess λ_max_ = 489 nm, *T*_g_ = 179 °C, *T*_D_ = 224 °C, and a large *d*_33_ coefficient 24.3 pm/V (1064 nm). Moreover, the NLO properties of this poled polymer exhibited long-term stability at 80 °C. A structurally similar chromophore incorporated into a sol–gel hybrid film, **145**, was investigated by Qian et al. (Figure 20; [105]). This thermally poled film showed λ_max_ = 487 nm, *T*_D_ = 272 °C, exceptionally high *d*_33_ = 42.0 pm/V, but no clear glass-transition behavior between 40–200 °C, because the rigid silica backbone hinders the motion of the molecule at higher temperature.

## Conclusion

This review has attempted to show that 1,3-diazole, imidazole, may act as a robust and stable parent π-conjugated backbone for organic chromophores with intramolecular charge transfer. This synthetically readily accessible five-membered heteroaromate and its push–pull derivatives are currently of high interest for materials chemists due to their unique and tunable properties. In general, the imidazole-derived chromophores may possess two Y-shaped arrangements: One electron donor at C2 and two electron acceptors at C4/C5 or vice versa. Hence, according to the C4/C5 substitution, the entire imidazole moiety may behave as an electron acceptor or donor. Taking our series of structurally similar chromophores **21**–**26** and **88**–**93** as an example, which primarily differ in the orientation of the substituents along the imidazole ring, C4/C5 donor-substituted imidazole derivatives showed higher nonlinearities. This implies that imidazole is more polarizable in the direction C4/C5→C2. However, two imidazole units that are differently C4/C5 substituted and connected at C2 may be employed as acceptor or donor moieties. It was shown that this diimidazole arrangement (e.g., in **101**–**111**) represents very powerful chromophore with high nonlinearities. Push–pull benzimidazoles feature more-planar π-conjugated systems due to the fused benzene ring. This fact further improves the polarizability of the entire D-π-A chromophore (e.g., compare chromophores **5**–**8** with **37**–**40**). The structure and the length of the π-linker connecting both acceptor and donor moieties play a crucial role. It was shown that polarizable subunits, such as olefins and thiophenes, increase the chromophore (hyper)polarizability significantly. Thus, the most important structural factors affecting D–A interaction responsible for the linear and nonlinear optical properties are (i) the strength of the appended donors and acceptors; (ii) the length and electronic nature of the π-conjugated path; and (iii) chromophore overall planarity. These three features mainly dictate the chromophore properties and, therefore, are mainly used to finely tune the desired (non)linearities. Imidazole-derived chromophores have found also a wide range of practical applications in OLEDs, OPVCs, switches, memories, and polymers. A combination of all of these properties makes imidazole a very promising scaffold for materials chemistry.
